# Quantitative dot blot analysis (QDB), a versatile high throughput immunoblot method

**DOI:** 10.18632/oncotarget.17236

**Published:** 2017-04-19

**Authors:** Geng Tian, Fangrong Tang, Chunhua Yang, Wenfeng Zhang, Jonas Bergquist, Bin Wang, Jia Mi, Jiandi Zhang

**Affiliations:** ^1^ Medicine and Pharmacy Research Center, Binzhou Medical University, Yantai, P. R. China; ^2^ Yantai Zestern Biotechnique Co. LTD, Yantai, P. R. China; ^3^ Department of Chemistry, BMC, Uppsala University, Uppsala, Sweden

**Keywords:** quantitative dot blot, high throughput, proteomics, immunoblot analysis, western blot

## Abstract

Lacking access to an affordable method of high throughput immunoblot analysis for daily use remains a big challenge for scientists worldwide. We proposed here Quantitative Dot Blot analysis (QDB) to meet this demand. With the defined linear range, QDB analysis fundamentally transforms traditional immunoblot method into a true quantitative assay. Its convenience in analyzing large number of samples also enables bench scientists to examine protein expression levels from multiple parameters. In addition, the small amount of sample lysates needed for analysis means significant saving in research sources and efforts. This method was evaluated at both cellular and tissue levels with unexpected observations otherwise would be hard to achieve using conventional immunoblot methods like Western blot analysis. Using QDB technique, we were able to observed an age-dependent significant alteration of CAPG protein expression level in TRAMP mice. We believe that the adoption of QDB analysis would have immediate impact on biological and biomedical research to provide much needed high-throughput information at protein level in this “Big Data” era.

## INTRODUCTION

Lacking of an accessible high throughput immun-oblot method significantly hinders any attempts to investigate the molecular basis of biological and pathological processes systematically in an average research lab. Until now, Western blot analysis remains the most commonly used immunoblot tool in the basic research lab almost 40 years since its invention [1–4]. However, its limitations, including complicated processing steps, limited ability to process many samples, ambiguity in result analysis and requirement of large amount of total protein lysate for analysis, determines this technique an unlike choice for high throughput analysis. As an alternative, Dot blot analysis was developed to simplify the process of Western blot analysis [5]. In fact, both enzyme-linked immunosorbent assay (ELISA) [6–8] and reverse phase protein microarray (RPPM) [9, 10] can be considered as Dot Blot analysis in a high throughput format. Nonetheless, the applications of these techniques are still limited in basic research lab for various reasons, including limited availability for prefabricated ELISA kits and lack of easy access to RPPMs.

**Figure 1:**
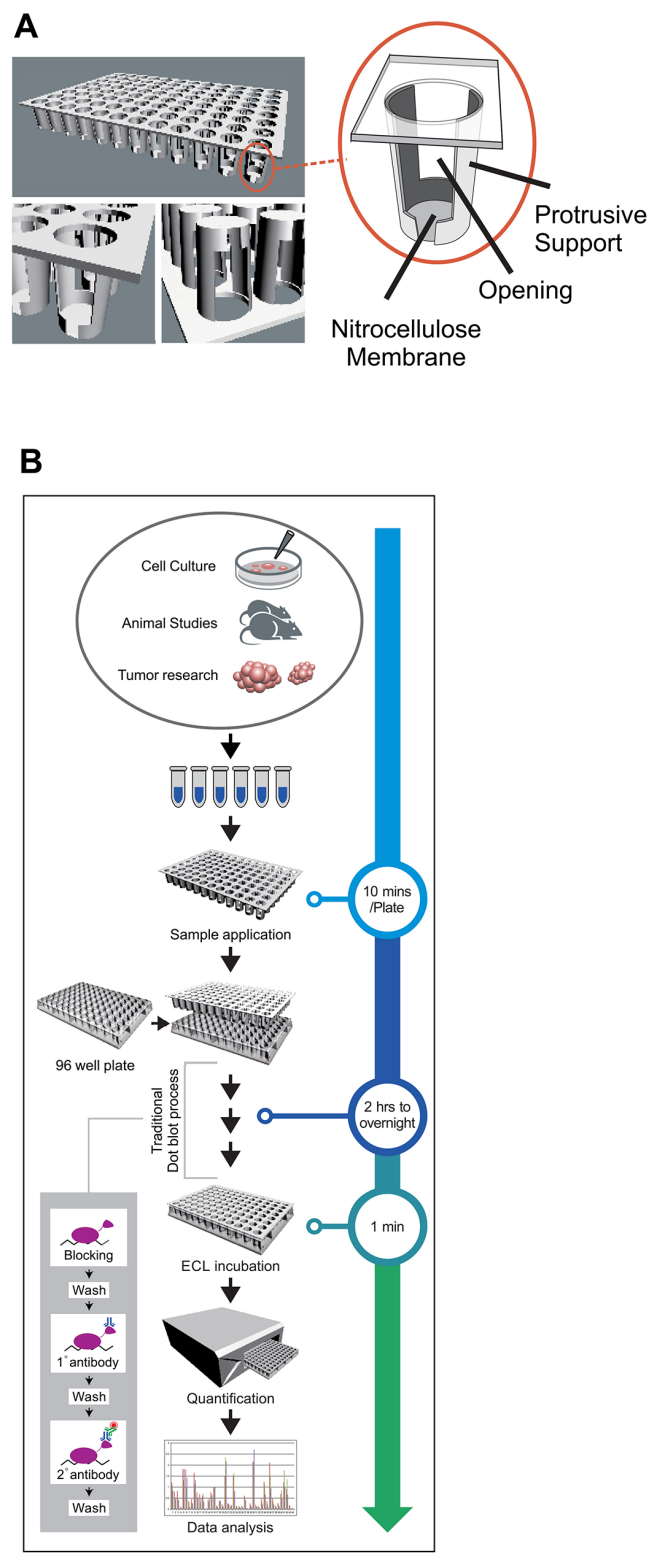
QDB analysis, concept and evaluation **(A)** An illustration of a QDB plate. QDB plate is made of a multi-unit plate of protrusive ends attached with a nitrocellulose membrane. A detailed illustration of an individual unit of QDB plate is shown in the enlarged portion. There are openings on both sides of the individual wells to facilitate the exchange of the solution during incubation and washing steps. **(B)** an illustration of the process of QDB analysis with estimated time required for each step.

Meanwhile, with the rapid development of effective high-throughput tools in genetic research, there are strong demands for complementary high throughput immunoblot methods on a daily basis for biomarker identification and other association studies at protein level [11, 12]. We have now developed a novel immunoblot method as convenient and robust as the traditional dot blot analysis, yet in high throughput format to meet this demand. We reasoned that achieving direct quantification of individual dots in the traditional dot blot analysis, rather than through an extra image conversion process, should significantly improve upon the traditional method. In order to achieve this goal, we have introduced a novel multi-unit plate in our analysis (Figure 1A), and named this method as Quantitative Dot Blot analysis (QDB), and the plate as QDB plate. The feasibility of this method was tested here at both cellular and tissue levels. Our results suggest that QDB analysis is able to produce reliable results with unprecedented efficiency and significant savings in research resources and efforts in an average research lab. It is also able to transform the current semi-quantitative immunoblot method into a true quantitative assay.

## RESULTS AND DISCUSSION

The typical QDB process is illustrated in Figure 1B (a detailed instruction of QDB analysis was included as Supplementary Data). The samples are applied directly to the membrane bottom of the individual units. The loaded membrane is then processed through steps of traditional immunoblot analysis to form immunocomplexes on the membrane. At detection step, the QDB plate is developed through a chemiluminescence reaction, and a white microplate is used as support to accommodate QDB plate in a microplate reader for quantification. To ensure a valid result, the specificity of the applied antibodies needs to be verified through Western blot analysis.

**Figure 2:**
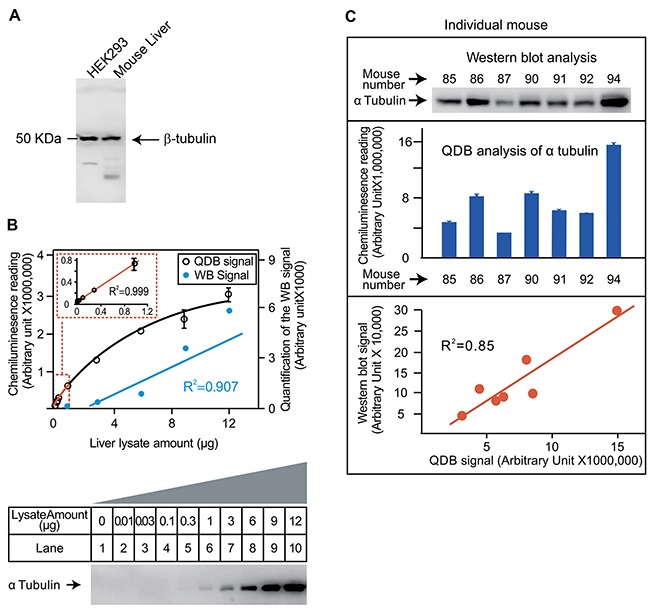
Evaluation of the feasibility of QDB analysis using an anti-tubulin antibody **(A)** Evaluation of the specificity of the antibody. HEK293 whole cell lysate and mouse liver lysate were prepared as described in the Materials and Methods. Lysates of 50μg/lane was used for Western blot analysis using an anti-tubulin antibody. The whole membrane was scanned using a blot scanner from Li-Cor. **(B)** Defining the linear range of QDB method and its comparison with Western blot analysis. Mouse liver lysate was serially diluted as indicated in the figure. The same amount of liver lysate was used for both Western blot analysis and QDB analysis side by side. In the upper panel, the QDB analysis of the dose curve of tubulin content in liver lysate is shown, with the linear region of the curve re-plotted as the insert in the figure. In the lower panel, the result of Western blot analysis is shown. This image is converted digitally using Image Studio Digits from Li-Cor (Lincoln, NE, USA), and plotted with the result of QDB analysis in the upper panel for comparison purpose. **(C)** Evaluation of the consistency of QDB analysis with Western blot analysis. Upper panel, Western blot analysis of tubulin contents in mouse livers. Liver slices of roughly similar size from 7 mice were homogenized as described in the Materials and Methods. Equal volume of liver lysate (20 μL, representing about 40 μg total protein/lane) from each mouse was used for the Western blot analysis. Middle panel, QDB analysis of tubulin content in mouse livers. The same lysates prepared in the upper panel were diluted and loaded to the individual unit of the QDB plate at 2 μL/unit or about 1 μg total protein/unit in triplicate. The result is the average of the triplicate from each mouse ±SEM. The results of Western blot analysis and QDB analysis using lysate from the same mouse were aligned to each other for comparison purpose. At lower panel, quantified result of the Western blot analysis using Image Studio Digits from Li-Cor was used to plot against that of the QDB analysis. The simple linear regression analysis was performed with R^2^ as 0.85. Mouse number refers to the assigned number of individual mice for recordkeeping.

The feasibility of QDB analysis was tested first by analyzing the tubulin content in mouse liver lysates. The specificity of a rabbit anti-tubulin antibody was evaluated in Figure 2A. For dose curve study, we serially diluted lysate from 0.01μg to 12 μg using pooled lysate prepared from 4 mouse livers. QDB analysis yields a linear curve between 0 to 1μg with coefficient of determination (R^2^) of 0.999 when simple linear regression analysis was performed (Figure 2B, upper panel). Beyond 1 μg, the signal started to reach plateau. In the same study, we could not detect visible signals until 1μg or more of lysate were used in Western blot analysis (Figure 2B, lower panel). Next, we evaluated the results from QDB analysis and Western blot analysis using individual mouse liver lysates prepared from 7 mice. The image from Western blot was quantified and compared with the result from QDB analysis. We were able to obtain R^2^ of 0.85 when simple linear regression analysis was performed on these two methods (Figure 2C). The inter- and intra-plate coefficient of variance (CV) of the QDB plate were also evaluated using the same antibody, with inter-plate CV at as lower as 4.37% and intra-plate CV at as lower as 5.19% (Supplementary Table 1). We also serially diluted samples prepared from mouse liver, and explored dose curves of several other antibodies. Our results showed that the linear range of the assay was highly dependent on the antibody *per se*. A further combination of biotin system and reporter enzyme could significantly increase the sensitivity of the assay. For most antibodies, over 20 fold of signal intensity over background can be detected when 0.25 μg to 2 μg sample lysates were used (Supplementary Figure 1).

Next, the feasibility of the QDB analysis was evaluated at cellular level by measuring the p65 (NF-κB p65 subunit) expression in stable clones from cells transfected with an RNAi plasmid against p65. We generated p65 stable clones by screening HEK293 cells transfected with a ShRNA plasmid against p65 (ShRNA-p65). Luciferase clones were generated using ShRNA-luciferase as negative controls. Total of 76 clones (71 p65 clones and 5 luciferase clones) were isolated after antibiotic selection.

**Figure 3:**
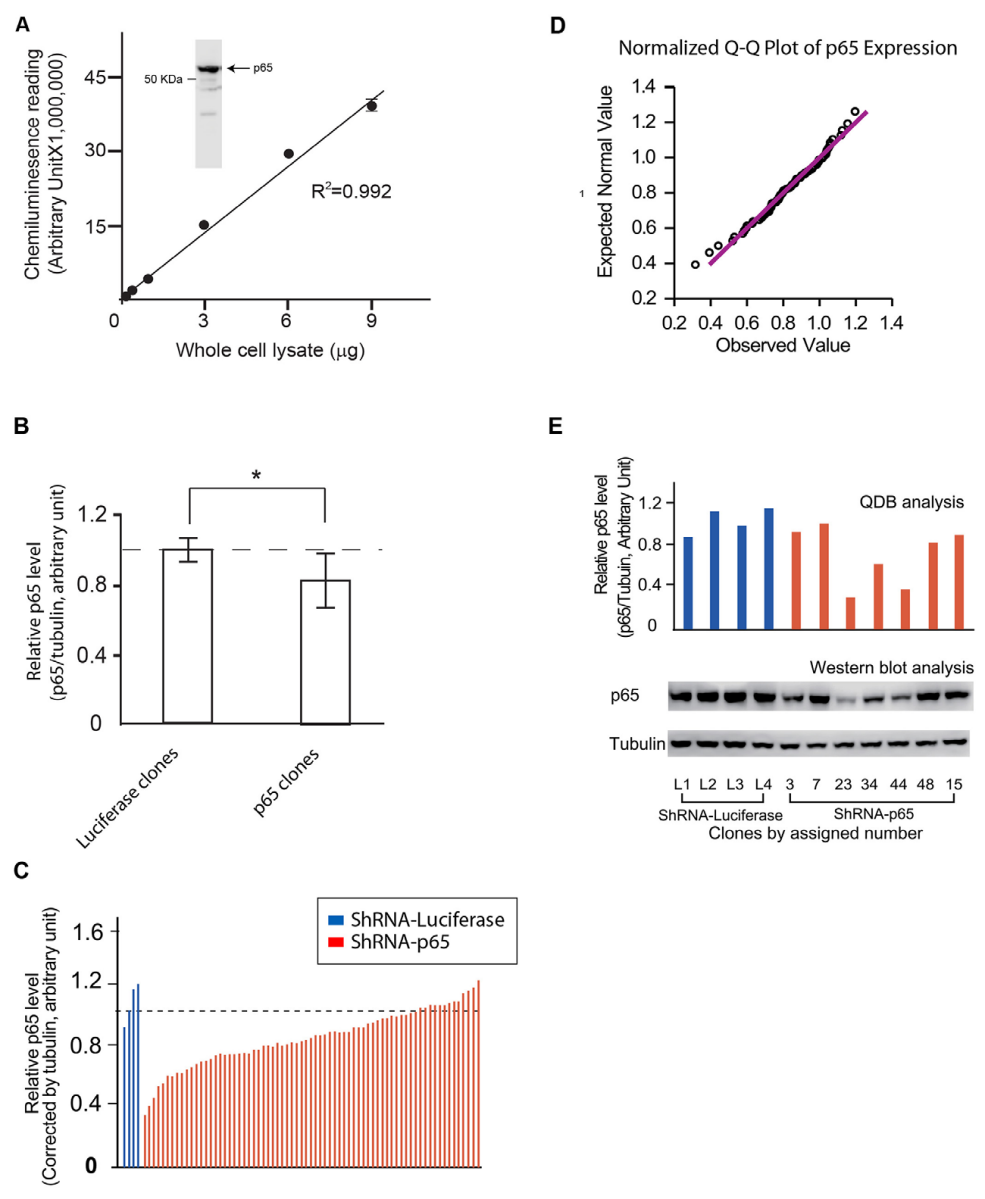
Evaluation of the feasibility of QDB analysis at cellular level **(A)** Characterization of anti-p65 antibody. Insert, the specificity of anti-p65 antibody was investigated using cellular lysate prepared with HEK293 cells in Western blot analysis. The whole membrane was scanned using a blot scanner from Li-Cor to ensure only one band with expected molecular weight was observed. A. The whole cell lysate was serially diluted as indicated in the figure, and applied to the QDB plate at 2μL/unit. The plate was processed as described in the Materials and Methods, and the results were analyzed with simple linear regression analysis with R^2^ at 0.992. **(B)** Comparison of the relative p65 levels between Luciferase and p65 clones. HEK293 cells were transfected with ShRNA-p65 or ShRNA-Luciferase respectively using Fugene 6 transfection reagent. Stable clones were selected using puromycin at 5 μg/mL from cells transfected with ShRNA-p65 (p65 clones) or ShRNA-Luciferase (Luciferase clones) until they were visible under naked eyes. Clones were picked up and transferred to two 96 well plate at 1:9 ratio (plate A and B for description purpose). Luciferase clones were labeled as L1 to L5, while p65 clones were labeled sequentially. The 96 well plates with a larger portion of cells (plate A) were allow to grow for one or two days before they were collected to prepare for cell lysate as described in the Materials and Methods. The whole cell lysate from individual clone was used for QDB analyses of tubulin and p65 levels, and the relative level of p65 (ratio of p65 level over tubulin level) was used to compare endogenous p65 expression levels in each clone, using the average of the p65 expression levels in luciferase clones as 1. The results were averaged to compare endogenous p65 levels between luciferase and p65 clones at population level. *, p< 0.05 using student T-test. **(C)** The relative p65 level of all 76 clones, including 5 luciferase clones and 71 p65 clones, was plotted individually. The result presented is the average of three independent experiments in triplicates. **(D)** The QQ plot analysis of the distribution of the endogenous p65 levels of 71 p65 clones is shown to demonstrate the normal distribution of relative p65 level among p65 clones. **(E)** Representative clones were picked up from the plates with less cells (plate B) based on the results shown in Figure 3C, and transferred to 60 mm dishes to allow them to reach sufficient cell numbers for Western blot analysis using anti-p65 and anti-tubulin antibodies (Lower panel). The results of QDB analyses of these representative clones from Figure 3C were re-plotted in the upper panel for comparison purpose.

The specificity and the application range of an anti-p65 antibody were evaluated in Figure 3A using whole cell lysate prepared from HEK293 cells. Next, the QDB analyses of the expression levels of both p65 and tubulin were evaluated, and the relative p65 expression level was determined by normalizing the p65 expression level of each p65 clone with its matching tubulin level. For comparison purpose, the average of the relative p65 levels of 5 luciferase clones was arbitrary set to 1, and the relative p65 level of each p65 clone was adjusted accordingly. As shown in Figure 3B, collectively, the p65 expression level showed significant difference between luciferase clones and p65 clones (p< 0.05 using student t-test). A detailed examination of the relative p65 expression level of each individual clone showed that while p65 expression levels were relative constant among the 5 luciferase clones, they displayed a wide range distribution among in p65 clones (Figure 3C). Interestingly, the q-q plot analysis indicated that the p65 expression levels followed a normal distribution at population level (Figure 3D). To the best of our knowledge, this is the first evaluation of cellular response to RNAi exposure at population level.

Currently, Western blot analysis is the most frequently used method for screening stable clones. However, screening of 76 clones in triplicate using Western blot analysis would be a formidable task. Therefore, we benchmarked our QDB result with that of Western blot analysis by picking 11 representative clones. These clones were allowed to grow to sufficient number of cells to provide enough amounts of total cell lysates for Western blot analysis. Our results indicated a high consistency between these two methods (Figure 3E).

It is worthy of mentioning that the advantage of QDB analysis over traditional immunoblot analysis is clearly demonstrated in this study. Traditionally, the picked clones needs to allow to grow at least 4 to 5 days either in 6 well plates or 60mm dishes before sufficient cell numbers can be reached to perform any immunoblot analyses. In contrast, the QDB analysis can be performed directly in 96 well plates after the isolation step to identify the target clones.

It is also conceivable that with QDB analysis, a lot of cellular studies can be performed conveniently with less time and resources when multi-well plates, rather than tissue culture dishes and flasks, are used in the research. For reference, we measured the yield of total protein amount from individual well of different types of multi-well plates using HEK293 cells. Indeed, more than 10 μg total cell lysate can be obtained from a single well of a 96 well plate, which could translate into 10 independent QDB analyses (Supplementary Table 2).

**Figure 4:**
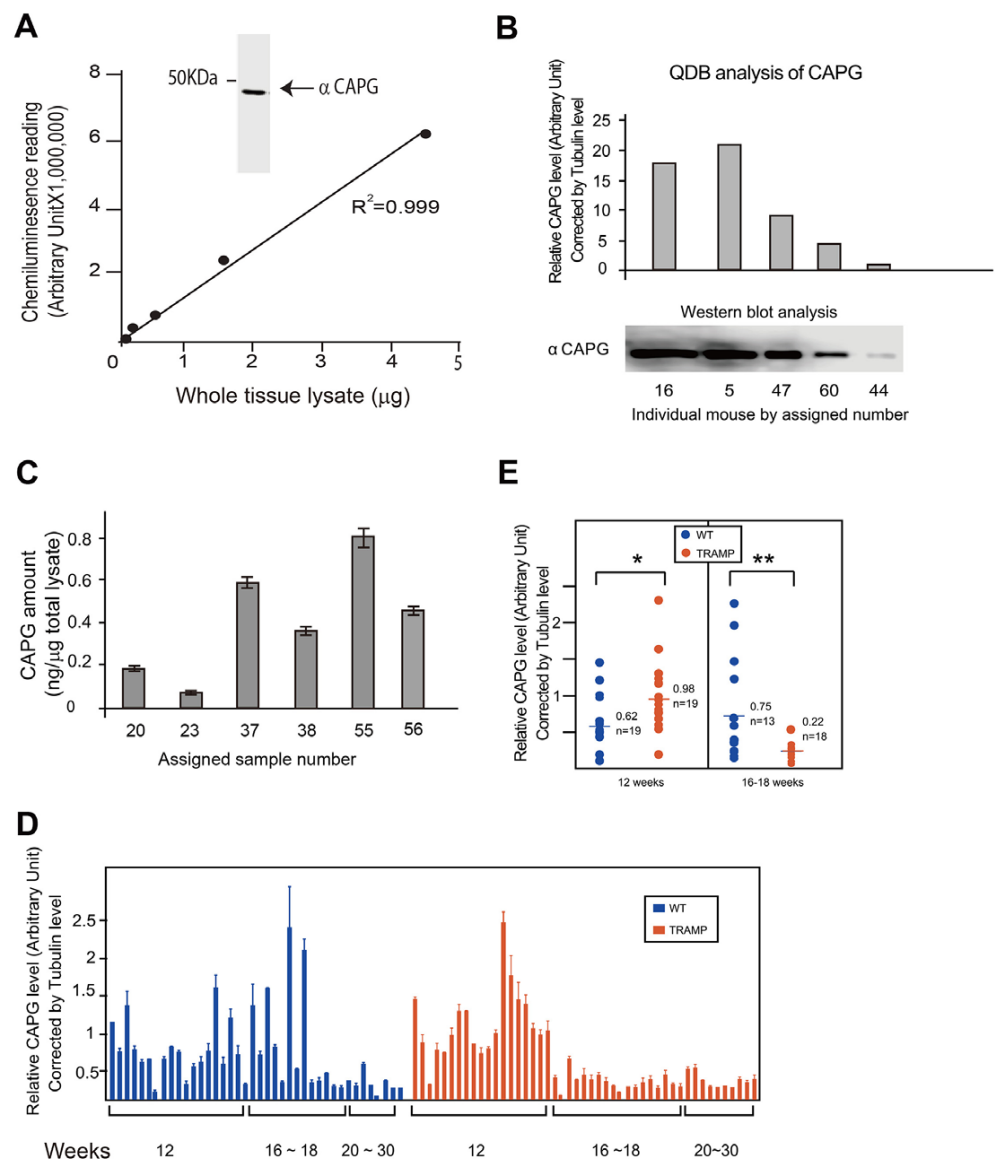
Evaluation of the feasibility of QDB analysis at tissue level **(A)** Characterization of Anti-CAPG antibody. Insert, Western blot analysis of anti-CAPG antibody. Mouse prostate tissue lysates were prepared as described in Materials and Methods. Prostate tissue lysates from 6 mice were mixed with equal amount based on BCA measurements, and 40 μg pooled total tissue lysate was used for Western blot analysis. In Figure A, serially diluted pooled mouse prostate tissue lysate was used to define the linear range of the QDB analysis at 2 μL/unit in triplicate. The results were analyzed with simple linear regression analysis with R^2^ at 0.999. **(B)** Benchmarking the QDB analyses of relative CAPG levels with Western blot analysis. Prostate tissues were collected from individual mice, as indicated by the assigned number, and whole tissue lysates were prepared as described in the Materials and Methods. For QDB analysis, prostate tissue lysates of 2 μL (around 1 μg total protein lysate per sample) in triplicate were used for the measurement of both tubulin and CAPG levels in individual mice. The relative CAPG level of individual mice, expressed as the ratio of CAPG level over tubulin level, is shown in the upper panel. In the lower panel, the amount of lysate of each sample used for Western blot analysis was adjusted based on the result of QDB analysis of tubulin levels of these samples to allow equal loading, and CAPG levels in these samples were examined using Western blot analysis. For lysate prepared from mouse #44, about 30 μg total tissue lysates were used. In the insert, the Western blot result from the lower panel was quantified using Image Studio Digits from Li-Cor. **(C)** Measuring the absolute amount of CAPG level in mouse prostate tissues. A dose curve was established using recombinant CAPG protein in Supplementary Figure 3. Samples prepared from prostate tissues of individual mice #20,23,37,38,55 were analyzed side by side in triplicate with the dose study in QDB analysis, and the absolute amount of CAPG were calculated based on the established dose curve. The results were average of 3 independent experiments ± SEM. **(D)** Prostate tissues from a total number of 87 mice (40 WT, 47 TRAMP) were used to prepare prostate tissue lysates as described in Materials and Methods. The protein concentration was measured by BCA protein determination kit. Total tissue lysates of 3 μl per sample in triplicate (about 1 μg total protein/unit) were used for each mouse for the QDB analyses of CAPG and tubulin levels. The result was plotted by age and phenotype. The results presented are averages of three experiments with each sample in triplicate in each experiment. **(E)** Comparison of relative CAPG levels by age group in wild type and TRAMP mice. The results were the averages of 19 WT mice and 19 TRAMP mice among 12 weeks old mice and averages of 18 WT mice and 13 TRAMP mice among mice of 16-18 weeks old. *, p<0.05, **, p< 0.01 based on student t-test.

Next, the QDB method was evaluated at tissue level by investigating the protein profile of Macrophage-capping protein (CAPG) in prostates from Transgenic Adenocarcinoma of the Mouse Prostate (TRAMP) mice and their wild type littermates (WT). CAPG is a protein involved in the metastasis of tumors, including prostate cancer [13–15]. After establishing the specificity and application range of the anti-CAPG antibody in QDB analysis (Figure 4A), we performed a pilot experiment to benchmark our analyses of the relative CAPG expression level (normalized against the tubulin expression level) against Western blot analysis in mouse prostate tissue (Figure 4B). Indeed, we were able to obtain R^2^ of 0.96 with simple linear regression analysis of these two methods (Supplementary Figure 2). To measure absolute amount of CAPG in mouse prostate tissue, a dose curve was established using recombinant CAPG protein directly with R^2^ at 0.994 in simple linear regression analysis (Supplementary Figure 3). Based on this dose curve, we were able to establish the linear range of this assay from 3 pg to 600 pg. CAPG amount of samples from several mouse prostate tissues were determined in the same QDB analysis based on this established dose curve (Figure 4C).

A large scale QDB analysis (n=87, with 40 WT and 47 TRAMP mice) of samples prepared from prostate tissues were performed, and the relative CAPG levels were compared between WT and TRAMP mice (Supplementary Figure 4). We were unable to observe any statistical differences in CAPG expression between these two groups collectively either based on the relative tubulin level (p=0.6766, Supplementary Figure 4A) or by absolute protein amount measured by BCA method (p=0.6959, Supplementary Figure 4B). However, when stratified with age, we were able to observe substantial variations of relative CAPG levels within both WT and TRAMP mice based on tubulin content. In addition, the relative CAPG level was age-dependent, with its expression level lowered in older mice. The overall profile of relative CAPG levels between WT and TRAMP group is also quite different from each other (Figure 4D).

These observations prompt us to further analyze our results by age group. As expected, the relative CAPG level of TRAMP mice was significantly higher at 12 weeks (p<0.05), and significantly lower at 16-18 weeks (p<0.01) compared to their WT littermates (Figure 4E). Our results thus demonstrate an age-dependent CAPG expression profiles with clear distinction between WT and TRAMP mice. While the biological significance of these results remains to be explored, technically these results would be hard to achieve with Western blot analysis alone. The substantial variations of CAPG level within each group also emphasizes the necessity of including more samples to better reflect and understand the complicated biological processes at protein level in animal and human studies.

Through these studies, we present a high throughput immunoblot method with the necessary qualities to be adopted in the research lab instantly. Maybe more importantly, we are able to transform the traditional immunoblot analysis into a true quantitative assay. The combined efforts to define the linear range of the assay by serial dilution of the sample and to direct quantify the signal through machine reading in QDB analysis also allow us to eliminate the ambiguity and bias associated with the image-conversion process in traditional immunoblot techniques.

QDB analysis can be ready transformed into an ELISA analysis when the purified protein, rather than the serially diluted sample is used to define the linear range of the analysis. The adoption of nitrocellulose membrane as the binding surface in QDB analysis significantly improves the binding capacity while reduces the background of the analysis for this method to be readily adopted in the research lab. In contrast, although ELISA is a quantitative high throughput method, the extensive developing efforts and costs prevent its routine application in the research lab.

QDB is distinct from traditional high throughput immunoblot techniques like ELISAs and RPPAs in multiple aspects. Compared with ELISAs, QDB method is more flexible and convenient. It requires less developing efforts and is easily adapted to samples from any new sources. In contrast, the validity of ELISA analysis is limited to sample from tested sources. Nonetheless, high quality ELISA kits had been subjected to strict and consistent pre-testing before they are available to the market. The sandwich-type ELISAs also have clear advantage in analyzing samples containing a lot of endogenous antibodies like plasma samples.

On the other hand, RPPAs has clear advantage when the total amount of samples is limited. It is also more convenient to process RPPA slides than QDB plates. Clearly, RPPAs have more advantage in analyzing complex signaling transduction pathways than QDB method. However, the requirements of sophisticated equipment and well-trained technicians in RPPA limit its accessibility to ordinary scientists. The semi-quantitative nature of RPPA analysis also demands further verification of its results independently. In fact, QDB method complements RPPAs well in this regard.

As demonstrated above, the QDB analysis is a convenient, affordable, versatile, quantitative, reliable and robust method. With its reliability and accuracy and its significant saving in research resources, we believe that this technique will find its application in many areas of biological and biomedical research including association studies at protein level. The adoption of this method may have an immediate impact on life science research with a lot more to promise in the near future.

## MATERIALS AND METHODS

### Reagents and animals

All general reagents for cell culture related work were purchased from Thermo Fisher Scientifics (Waltham, MA, USA) including cell culture medium and culture dishes. HEK293 cells were purchased from the Cell Bank of Chinese Academy of Sciences, Shanghai, China. QDB plate was manufactured by Yantai Zestern Biotechnique Co. Ltd, in Yantai, China. The protease inhibitors were purchased from Sigma Aldrich (St. Louis, MO, USA). All other chemicals were purchased from Sinopharm Chemicals (Beijing, P. R. China). Pierce BCA protein assay kit was purchased from Thermo Fisher Scientifics (Waltham, MA, USA). Recombinant human CAPG protein (14213-HNAE) was purchased from Sino Biological Inc. (Beijing, P. R. China).

### Mouse Strains

TRAMP mice and their wild type littermates were purchased from Jackson Laboratory (www.jax.org). These mice were from a C57BL/6 origin and obtained from C57- x C57-matings. Animals were supported under a 12/12 hours light-dark cycle with natural drink and food. All animal procedures were approved by the ethical review board of Binzhou Medical University (ER #2016-19). The genotype of animals and confirmation of tumorigenesis were described elsewhere [16, 17].

### Antibodies

Rabbit anti-tubulin (YT-0183), rabbit anti-ApoE (YT-0273) antibodies were purchased from Immunoway, Suzhou, P. R. China, Rabbit anti-p65 (SC-372, C20, F0414), Rabbit anti-CDK4 (sc-260, c22, A0314) were purchased from Santa Cruz Biotechnology, Inc. (Santa Cruz, CA, USA). Rabbit anti-CAPG (14213-T52), rabbit anti-ANXA6 (11161-T60), and rabbit anti-CALR (13539-T60) were purchased from Sino Biologic Inc. Beijing, P. R. China.

### Generation and Screening of stable clones

For construction of constitutive expressed RNA interference (RNAi) constructs, pGreenpuro plasmid from System Biosciences Inc. (Palo Alto, CA, USA) was used by following the manufacturer's instructions using a targeting sequence 5’GGACATATGAGACCTTCAAGA 3′ against p65 to create ShRNA-p65 plasmid or target sequence 5’GTGCGTTGTTAGTACTAATCCTATTT3’ against luciferase to create ShRNA-Luciferase plasmid.

ShRNA-p65 and ShRNA-Luciferase plasmids were used to transfect HEK293 cells at 5×10^5^/dish in two 60 mm dishes respectively using Fugene 6 transfection reagent by following manufacturer’ instructions. Cells were allowed to grow for two days in growth medium (DMEM medium supplemented with 10% fetal bovine serum) before they were changed into fresh selection medium (growth medium supplemented with 5μg/mL puromycin). The selection process continued by exchanging the selection medium every 3 to 4 days until visible clones could be seen with naked eyes. Individual clones were picked up by trypsin digestion using Cloning cylinder from Sigma, transferred to two parallel 96 well plates at 1:9 ratio, and labeled by the same clone number with plate A for 96 well plates with more cells, while B for those with less cells. The cells were allowed to growth 1 or 2 days in selection medium in plate A before total cell lysates were prepared by adding lysis buffer (50 mM Hepes, pH 7.4, 137 mM NaCl, 5 mM EDTA, 5 mM EGTA, 1 mM MgCl_2_, 10 mM Na_2_P_2_O_7_, 1% Triton X-100, 10% glycerol, supplemented with protease and phosphatase inhibitors (100 mM NaF, 0.1 mM phenylmethylsulfonyl fluoride, 5 μg/mL pepstatin, 10 μg/mL leupeptin, 5 μg/mL aprotinin) to the plate directly. Total cell lysates were prepared by collecting supernatant after 5 mins of centrifugation, and sample buffers were added directly to the supernatant for QDB analysis.

For plate B, representative clones based on the result from QDB analysis of plate A were transferred to two 60 mm dishes at 2 × 10^5^/dish, and cells were allowed to growth 7 days in selection medium before they were harvested either for preparation of total cell lysate and measurement of protein concentration (see next section), or for storage in liquid nitrogen.

### Cell and tissue extractions

For HEK293 cells, cells were harvested and lysed in lysis buffer by pipetting up and down 50 times. Supernatants were collected after 5 min of centrifugation at 8000 × g, and protein concentration was determined by using Pierce BCA protein assay kit from Thermo Fisher before they were resuspended in sample buffer for Western blot analysis. For preparing tissue lysates from mouse livers and prostates, tissues were sliced into microcentrifuge tubes pre-aliquoted with 300 mL lysis buffer with protease inhibitors. Tissues were minced with a handhold tissue homogenizer for 1 minute before the microcentrifuge tubes were subjected to centrifugation at 8000 × g for 5 mins. The supernatant from each tube was collected for measurement of protein content using Pierce BCA protein assay kit from Thermo Fisher and for Western blot analysis.

### QDB analysis

Prepared total lysate from 0.1 μg up to 12 μg/unit, as indicated in the figure, was applied directly on individual membrane unit of the QDB plate. The plate was left either at 37°C for 15 mins or at room temperature for 45 mins to dry membrane completely. The plate was rinsed with TBST (137 mM NaCl, 2.7mM KCl, 20 mM Tris, pH7.4, plus 0.1% Tween-20) for 3 times, and blotted with blocking buffer (5% non-fat milk in TBST) in one container. The plate was incubated with primary antibody either overnight at 4°C or for 2 hour at room temperature in either one big container if the whole plate was blotted with same antibody, or into a 96 well plate with different antibodies in different wells. The plate was washed three times with TBST, and incubated again with the secondary antibody for 2 hour before the plate was washed again for three times with TBST. The plate was inserted into a 96 well plate loaded with 100 μL/well ECL substrate solution for 1 minute before it was inserted into a white 96 well plate for chemiluminescence signal quantification using a Tecan Infiniti 200 pro microplate reader with the option “plate with cover” chosen in the user interface.

To reduce the inter-plate variations using QDB method, we pooled a reference sample by mixture 4 or 5 samples together at equal amount, and serially diluted this reference sample. A dose curve based on this reference sample was repeated in every plate to be analyzed, and was used to adjust the readings of each sample in the same plate accordingly.

### Statistical analysis

Data were presented as mean ± SEM, and analyzed with the two-tailed Student's t-test between two groups. The q-q plot was performed with SPSS v22.0 (IBM, Chicago, IL).

Note: Plates are available upon request for verification purpose either through email or visiting www.zestern.net.

## SUPPLEMENTARY MATERIALS FIGURES AND TABLES







## Abbreviations

QDB: Quantitative dot blot analysis
ELISA: enzyme-linked immunosorbent assay
RPPM: reverse phase protein microarray
RNAi: RNA interference
CV: coefficient of variance
ShRNA-p65: ShRNA plasmid against p65
CAPG: capping protein
TRAMP: TRansgenic Adenocarcinoma of the Mouse Prostate
WT: type littermates
