# Supplementary material for: Quantitative dot blot analysis (QDB), a versatile high throughput immunoblot method
Source: Oncotarget. 2017 Apr 19;8(35):58553–62. doi: 10.18632/oncotarget.17236 (PMC5601674; doi:10.18632/oncotarget.17236)
Supplement: Supplementary file 1 [file oncotarget-08-58553-s001.pdf]

Quantitative dot blot analysis (QDB), a versatile high throughput immunoblot method

SUPPLEMENTARY FIGURES AND TABLES

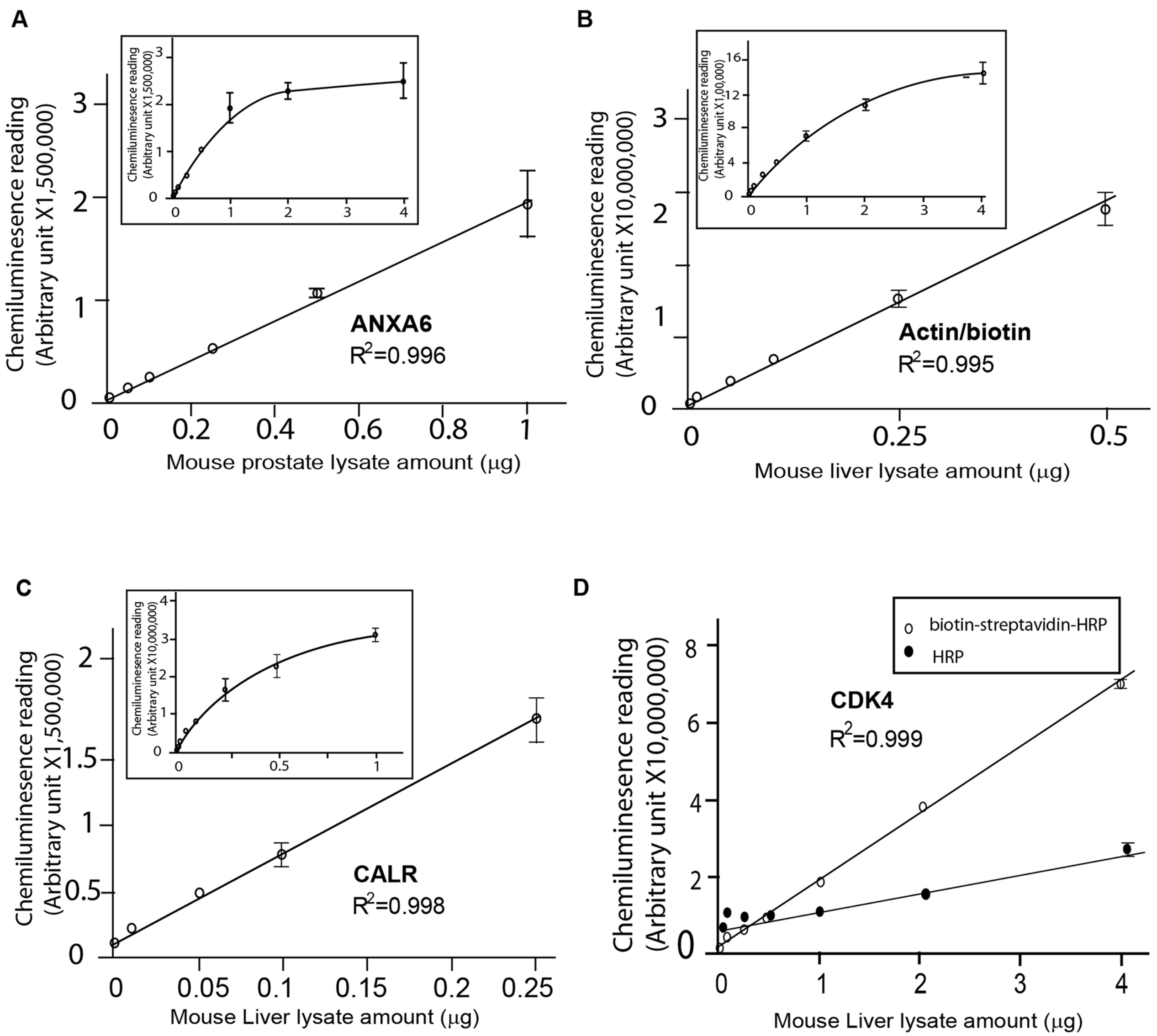

Supplementary Figure 1: Several dose curves of QDB analysis using either mouse liver lysates or prostate lysates. (A) linear range of QDB analysis of a mouse prostate lysate using anti-ANXA6 antibody. The insert is the overall dose curve ranging from 0.01 µg to 4 µg. **(B)** linear range of QDB analysis of a mouse liver lysate using anti-actin antibody. The signal was enhanced by coupling with a biotin-streptavidin system. The insert is the overal dose curve of the same lysate ranging from 0.01 to 4µg. **(C)** linear range of QDB analysis of a mouse liver lysate using anti-CALR antibody. The insert is the overall dose curve of the same lysate ranging from 0.01 to 1 µg. **(D)** linear range of QDB analysis of a mouse liver lysate using an anti-CDK4 antibody. The overall signals were compared with and without the enhancement of biotin-streptavdin system as indicated in the figure.

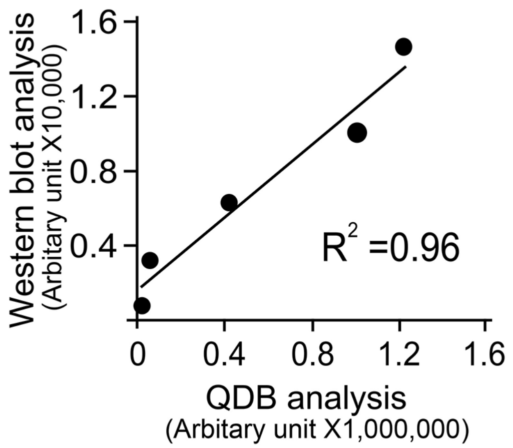

Supplementary Figure 2: The image of Western blot analysis in Figure 4B was quantified using Image Quant from Li-Cor, and the result was plottted against the result of QDB analysis. The correlation of these two methods was analysis using simple linear regression analysis with R =0.96.

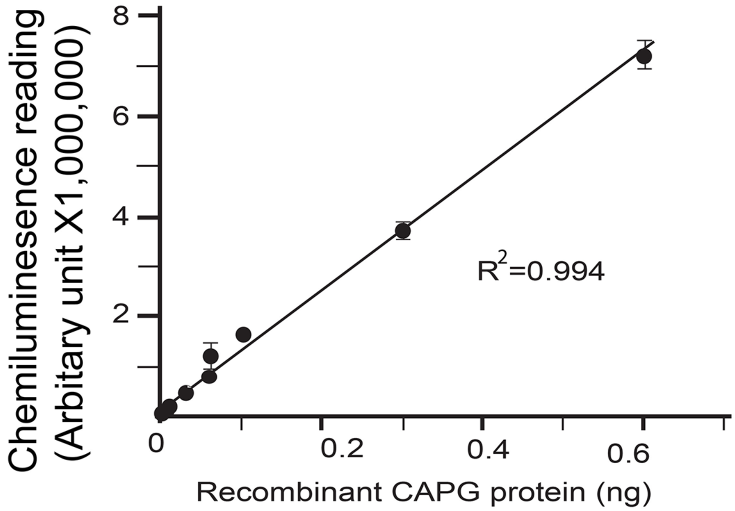

Supplementary Figure 3: Human recombinant CAPG protein was serially diluted as indicated in the Figure, and applied directly to the QDB plate at 2 µl/sample. QDB analysis was performed using recombinant protein side by side with mouse prostate tissue samples. The results were average of triplicate+SEM.

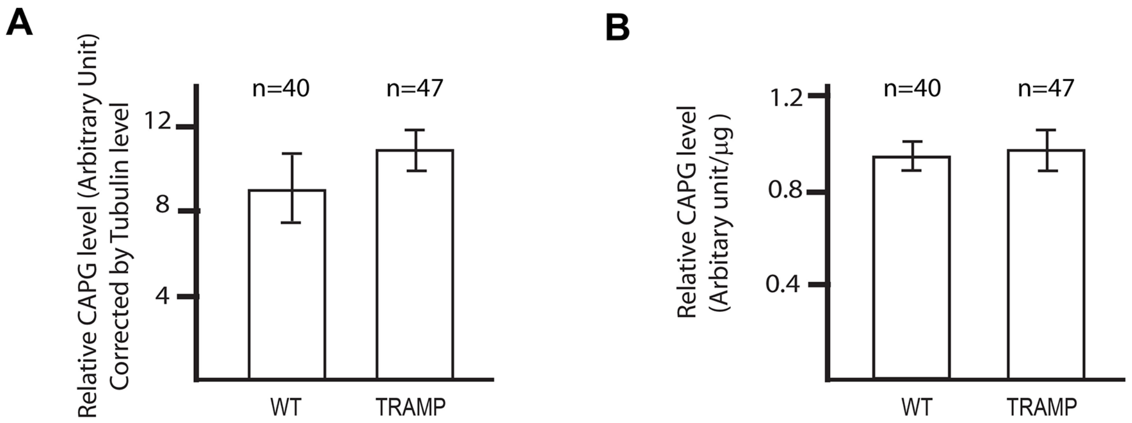

Supplementary Figure 4: Comparison of relative CAPG levels between TRAMP and WT mice. Prostate tissues from a total number of 87 mice (40 WT, 47TRAMP) were used to prepare prostate tissue lysates as described in Materials and Methods. The protein concentration was measured by BCA protein determination kit. Total tissue lysates of 3 µl per sample in triplicate (about 1 µg total protein/unit) were used for each mouse for the QDB analyses of CAPG and tubulin levels. The relative levels of CAPG of each mouse either by tubulin level **(A)** or protein amount **(B)** were used to determine the average relative CAPG level by genotype. In A, p= 0.6766 by student T-test. In B, p=0.6959 by student T-test.

Supplementary Table 1: Intra-plate and Inter-plate CV% of QDB plate.

See Supplementary File 1

Supplementary Table 2: Yield of the total lysate amount from individual well of different multi- well plates. HEK293 cells were seeded as indicated in the table in different types of multi-well plates. Cells were grown in DMEM plus 10% fetal bovine serum for 72 hours before total cells from individual wells were harvested. Cells were lysized in lysis buffer as described in the Materials and Methods, and total protein amount of the lysates were determined using BCA method by following the manufacturer's instructions.

	Cells seeded	Total protein amount after 72 hours (µg)
96 well plate	2.8X104/well	12.9
48 well plate	6.7X104/well	32.8
24 well plate	2.0X105/well	109.1

Supplementary Data: QDB Instruction

See Supplementary File 2
