# Supplementary material for: Quantitative dot blot analysis (QDB), a versatile high throughput immunoblot method
Source: Oncotarget. 2017 Apr 19;8(35):58553–62. doi: 10.18632/oncotarget.17236 (PMC5601674; doi:10.18632/oncotarget.17236)
Supplement: Supplementary file 2 [file oncotarget-08-58553-s002.pdf]

	Experiment	1	2	3	4	5	6	7	8	9	10	Inter-plate CV
Assigned mouse #												
blank		257923.00	204723.0	101611.00	76883.00	71662.00	63832.00	175870.00	268015.00	120922.00	129553.00	
		273368.00	180347.0	121251.00	81944.00	79463.00	64481.00		250070.00	111822.00	101549.00	
		266133.00	205961.0	108192.00			45037.00	165724.00	201396.00	114723.00	131913.00	
	average	265808.00	197010.3	110351.33	79413.50	75562.50	57783.33	170797.00	239827.00	115822.33	121005.00	
	stdev	7727.63	14444.1	9996.47	3578.67	5516.14	11043.42	7174.31	34470.45	4648.54	16890.66	
	%CV	2.91	7.3	9.06	4.51	7.30	19.11	4.20	14.37	4.01	13.96	8.68
83		4992204.00	5265429.0	4737686.00	3514563.00	4145321.00	3009564.00	3921988.00	4834340.00	5059482.00	5650137.00	
		5447068.00	5277402.0	5001807.00	4040884.00	4254870.00	2997768.00	4642965.00	4585008.00	4444240.00	5108010.00	
		4807332.00	4099196.0	4886265.00	3224871.00		3075140.00	4581605.00			4464380.00	
	average	5082201.33	4880675.6	4875252.67	3593439.33	4200095.50	3027490.67	4382186.00	4709674.00	4751861.00	5074175.67	
	stdev	329226.64	676807.7	132404.42	413685.16	77462.84	41684.90	399722.29	176304.35	435041.79	593602.13	
	%CV	6.48	13.8	2.72	11.51	1.84	1.38	9.12	3.74	9.16	11.70	7.15
84		9352025.00	11141365.0	10585928.00	8026155.00	8063921.00	6624539.00	10848321.00	9593500.00	9949815.00	10110061.00	
		10460581.00	10259501.0	10089027.00	6950462.00	7060666.00	5511988.00	10707858.00	9713867.00	10162348.00	11712526.00	
			10778940.0	8579763.00		7227813.00	6606523.00	9577128.00	9264168.00	9831606.00	12113207.00	
	average	9906303.00	10726602.0	9751572.67	7488308.50	7450800.00	6247683.33	10377769.00	9523845.00	9981256.33	11311931.33	
	stdev	783867.46	443255.5	1044787.57	760629.81	537515.16	637194.52	696923.22	232800.69	167597.69	1059955.44	
	%CV	7.91	4.1	10.71	10.16	7.21	10.20		2.44	1.68	9.37	7.09
85		6921935.00	7291694.0	7208681.00	5300625.00	4688162.00	4343827.00	6317790.00	6801831.00	6360800.00	7471324.00	
		6354484.00	6270059.0	7070935.00	5002961.00	5296768.00	3618828.00	6223924.00	6257508.00	5832360.00	7460264.00	
		6016568.00	5312430.0	7173791.00	5037291.00	5548477.00	4145393.00	5640935.00	5463434.00	5978355.00		
	average	6430995.67	6291394.3	7151135.67	5113625.67	5177802.33	4036016.00	6060883.00	6174257.67	6057171.67	7465794.00	
	stdev	457507.24	989804.4	71613.11	162853.31	442323.52	374671.03	366701.44	673071.02	272894.23	7820.60	
	%CV	7.11	15.7	1.00	3.18	8.54	9.28	6.05	10.90	4.51	0.10	6.64
86		11496452.00		13046830.00	8788833.00	10528691.00	7996112.00	12977436.00	11491188.00	11602874.00	13572050.00	
		11242174.00	13053215.0	12805279.00	8477063.00	10623064.00	7764398.00	11599689.00	11610982.00		12461320.00	
		10370962.00	12400717.0	12798948.00	7812246.00	9444800.00		12364316.00	12381634.00	12493640.00	12986196.00	
	average	11036529.33	12726966.0	12883685.67	8359380.67	10198851.67	7880255.00	12313813.67	11827934.67	12048257.00	13006522.00	
	stdev	590253.53	461385.7	141322.59	498815.99	654730.48	163846.54	690260.50	483244.10	629866.68	555643.90	
	%CV	5.35	3.6	1.10	5.97	6.42	2.08	5.61	4.09	5.23	4.27	4.37
87		5653344.00	5428436.0	5837550.00	3611708.00	4331900.00	3404892.00	5577983.00	5276137.00	5176022.00	6659764.00	
		5747982.00	5187147.0	5546210.00	4003733.00	4230046.00	3325398.00	5328506.00	5497367.00	5465650.00	6303515.00	
		4962280.00		5498592.00	3949731.00		3707959.00	4934722.00	4780849.00	5584030.00	6246835.00	
	average	5454535.33	5307791.5	5627450.67	3855057.33	4280973.00	3479416.33	5280403.67	5184784.33	5408567.33	6403371.33	
	stdev	428923.74	170617.0	183502.50	212469.36	72021.65	201875.30	324317.06	366890.30	209908.21	223843.82	
	%CV	7.86	3.2	3.26	5.51	1.68	5.80	6.14	7.08	3.88	3.50	4.79
88		4381043.00		4055386.00	2675349.00	3827788.00	2692040.00	5499243.00	5035632.00	4403539.00	5151930.00	
		4806735.00	4435202.0	4286525.00	3124200.00	3927183.00	3035641.00	4966158.00	4613121.00	3827837.00	5341939.00	
		4353950.00	4559729.0	3657597.00		4233011.00	2862757.00	5304366.00	4716800.00	3255295.00		
	average	4513909.33	4497465.5	3999836.00	2899774.50	3995994.00	2863479.33	5256589.00	4788517.67	3828890.33	5246934.50	
	stdev	253956.02	88053.8	318122.55	317385.59	211193.36	171801.64	269734.84	220196.40	574122.72	134356.65	
	%CV	5.63	1.9	7.95	10.95	5.29	6.00	5.13	4.60	14.99	2.56	6.51
89		8748742.00	9336499.0	11327895.00	6479851.00	6826095.00	6976274.00	9131935.00	7704992.00	8858538.00	9758482.00	
		9042091.00	9835071.0	10690956.00	6054366.00	8313501.00	6118803.00	9898596.00	8341177.00	7640989.00	10661228.00	
		8125953.00	8204143.0	9728878.00	7136080.00	7409280.00	5339339.00	8484868.00	8162023.00	8691494.00	10352047.00	
	average	8638928.67	9125237.6	10582576.33	6556765.67	7516292.00	6144805.33	9171799.67	8069397.33	8397007.00	10257252.33	
	stdev	467836.97	835736.2	804999.04	544943.29	749455.02	818777.22	707706.58	328051.02	660036.62	458777.84	
	%CV	5.42	9.1	7.61	8.31	9.97	13.32	7.72	4.07	7.86	4.47	7.79
90		11146284.00	12383479.0	14248959.00	9253049.00	11139971.00	6927604.00	13865610.00	13036882.00	13057173.00	11180771.00	
		11349525.00	11846242.0	12869377.00	8616935.00	12598864.00	9077586.00	14429219.00	12958677.00	12025374.00	13207785.00	
		12855211.00	10113107.0	12771333.00		8604518.00		12064310.00	10429314.00	10059617.00	12577823.00	
	average	11783673.33	11447609.3	13296556.33	8934992.00	10781117.67	8002595.00	13453046.33	12141624.33	11714054.67	12322126.33	
	stdev	933526.35	1186519.3	826260.42	449800.52	2021208.00	1520266.85	1235255.13	1483419.70	1522834.62	1037416.03	
	%CV	7.92	10.3	6.21	5.03	18.75	19.00	9.18	12.22	13.00	8.42	11.01
91		9331017.00	7664136.0	10412341.00	6238772.00	7828728.00	5271917.00	9153408.00	8648790.00	8108337.00	9716124.00	
		8971237.00	8778273.0	11431708.00	5982607.00	7989412.00	5238024.00	10513581.00	9332853.00	8565278.00	10381208.00	
		7553300.00	7213359.0	8613320.00	5927131.00	7035269.00	6203650.00	9143733.00	7217959.00		10855472.00	
	average	8618518.00	7885256.0	10152456.33	6049503.33	7617803.00	5571197.00	9603574.00	8399867.33	8336807.50	10317601.33	
	stdev	939881.62	805549.1	1427053.87	166241.90	510846.58	547982.46	788104.03	1079196.94	323106.08	572331.04	
	%CV	10.91	10.2	14.06	2.75	6.71	9.84	8.21	12.85	3.88	5.55	8.49
92		7379319.00	7786006.0	7133101.00	5385934.00	7356026.00	5271995.00	8480860.00	8874315.00	8011840.00	8656388.00	
		6871388.00	6346237.0	8384356.00	5317302.00	7814914.00	5283543.00	8101480.00	7050423.00	7000206.00	7447713.00	
		8242889.00	7329022.0	7510075.00	5246169.00	7451557.00	5353100.00	9692217.00	7183931.00	6795589.00	8259304.00	

	average	7497865.33	7153755.0	7675844.00	5316468.33	7540832.33	5302879.33	8758185.67	7702889.67	7269211.67	8121135.00	
	stdev	693392.87	735712.3	641887.29	69886.23	242120.04	43873.97	830838.85	1016677.96	651221.67	616069.65	
	%CV	9.25	10.2	8.36	1.31	3.21	0.83	9.49	13.20	8.96	7.59	7.25
93		9397191.00	8534946.0	9632597.00	6496924.00		6299626.00	6379096.00	8217390.00	8107002.00	10104783.00	
		10040737.00	8643472.0	10465941.00	6906026.00	7450459.00	5998831.00	9371880.00	7286649.00	6563313.00	10955497.00	
		7898586.00	7084078.0	9513505.00	5487646.00	8317192.00	6016125.00	9399736.00	7578588.00	8633963.00	10584588.00	
	average	9112171.33	8087498.6	9870681.00	6296865.33	7883825.50	6104860.67	8383570.67	7694209.00	7768092.67	10548289.33	
	stdev	1099149.62	870680.3	518937.93	730046.61	612872.78	168893.23	1735981.86	476020.87	1076123.83	426517.02	
	%CV	12.06	10.7	5.26	11.59	7.77	2.77	20.71	6.19	13.85	4.04	9.50
94		16583479.00	17925146.0	20867556.00	10431889.00	18389824.00	13345207.00	18556128.00	17850038.00	16676455.00	17338972.00	
		17330760.00	14174062.0	21099228.00	13849096.00	15358490.00	12553013.00	17897672.00	16457481.00	16147852.00	17797916.00	
		10224384.00	15846225.0	15503840.00	9916920.00	18510576.00	11997451.00	21072708.00	14789130.00	16237366.00	17640216.00	
	average	14712874.33	15981811.0	19156874.67	11399301.67	17419630.00	12631890.33	19175502.67	16365549.67	16353891.00	17592368.00	
	stdev	3905062.91	1879214.0	3165740.78	2137151.72	1786020.39	677331.37	1675688.56	1532523.40	282911.36	233183.34	
	%CV	26.54	11.7	16.53	18.75	10.25	5.36	8.74	9.36	1.73	1.33	11.03
95		11673902.00	9790503.0	12295580.00	6794674.00	9175328.00	6792898.00	11228940.00	7428758.00	9775306.00	11806924.00	
		9678571.00	10089710.0	11511060.00	7471402.00	9081886.00	7330957.00	10972328.00	7649987.00	8232731.00	10868093.00	
		10563695.00	9096960.0	12143638.00	6947876.00	8506920.00	6798697.00	9743707.00	9307125.00	7543860.00	10795686.00	
	average	10638722.67	9659057.6	11983426.00	7071317.33	8921378.00	6974184.00	10648325.00	8128623.33	8517299.00	11156901.00	
	stdev	999779.13	509260.7	416075.50	354849.99	361959.15	308988.09	793859.39	1026589.11	1142616.31	564099.39	
	%CV	9.40	5.2	3.47	5.02	4.06	4.43	7.46	12.63	13.42	5.06	7.02
96		2483478.00	2362862.0	3174477.00	1698955.00	1946718.00	1560927.00	2237978.00	2266585.00	2099497.00	2378985.00	
		2520295.00	2031147.0	2304654.00	1421040.00	1815193.00	1409855.00	2284871.00	2115447.00	1960800.00	2608093.00	
		2308750.00	1878963.0	1929951.00	1356590.00		1457940.00	2301700.00	2178156.00	2444008.00	2543518.00	
	average	2437507.67	2090990.6	2469694.00	1492195.00	1880955.50	1476240.67	2274849.67	2186729.33	2168101.67	2510198.67	
	stdev	113016.70	247437.8	638466.83	181936.04	93002.22	77180.78	33021.87	75932.87	248802.02	118132.35	
	%CV	4.64	11.8	25.85	12.19	4.94	5.23	1.45	3.47	11.48	4.71	
intra assay CV(n=15)		9.03	8.7	8.15	8.02	6.90	6.82	8.08	7.63	8.12	5.19	

Supplemental table 1: Intra-plate and Inter-plate CV% of Zestern plate.

Mouse livers were collected from 15 individual mice, indicated by the assigned number. Total liver lysates were prepared from these mouse livers and used to measure tubulin content using QDB analysis. Each sample was measured in triplicate, and the experiment was repeated 10 times in 2 weeks. The intra and inter plate CV% were calculated by following the method introduced at the following website: https://www.salimetrics.com/assets/documents/Spit_Tips_-_Inter__Intra_Assay_Coefficients_of_Variability.pdf.

For 15 samples in 10 repeats, the interplate CV% is ranging from 4.37% to 11.03%. For 15 samples in each experiment, the intra-plate CV% is ranging from 5.19% to 9.03%.
