# Supplementary material for: Quantitative dot blot analysis (QDB), a versatile high throughput immunoblot method
Source: Oncotarget. 2017 Apr 19;8(35):58553–62. doi: 10.18632/oncotarget.17236 (PMC5601674; doi:10.18632/oncotarget.17236)
Supplement: Supplementary file 3 [file oncotarget-08-58553-s003.pdf]

QDB operational instruction

I. Reagents

1. Transfer buffer (For 1L):

   3g Tris base

   14.4 g Glycine

   700 mL ddH2O

   200mL Methanol, adjust to 1L

2. 10x TBS (For 1L):

   24.2g Tris base

   80g NaCl

   Bring up the volume to 0.8L with ddH2O

   Adjust the pH to 7.6 with concentrated HCl

   Bring up the volume to 1L with ddH2O

3. TBST (For 1L):

   100 ml 10X TBS, Add 10 mL 10% Tween-20 with 1L dd H_2O

4. Blocking buffer (For 100 mL):

   4g nonfat milk powder

   90mL TBST

   Bring up the volume to 100mL with TBST.

II. Procedure:

1. **Sample preparation**. Sample can be prepared using various lysis buffers developed for Western blot. The protein amount can be determined either through protein

concentration determination kits like Bradford or BCA, or by the weight or cell numbers. The lysates are resuspended in sample buffer with DTT, and heated at 85 C for 5 mins.

2. **Determination of antibody specificity**. Roughly equal amount of prepared samples (based either by cell number, weight, or protein concentration) from 4 or 5 samples are mixed well, and used for Western blot analysis. The specificity of the antibody is demonstrated by showing one band of right size on the Western blot analysis.
3. **Defining the linear range of QDB analysis**. The mixture used for Western blot analysis is also used to establish the linear range of the QDB analysis by serial dilution of the mixed samples from 0.03 μg to 9 μg in equal volume. Concentrated IgG free BSA solution could be used to supplement the diluted sample to allow equal amount of protein loading on the membrane.
4. **Sample application**: Place the QDB plate on an empty pipetman tip box to avoid the bottom of the plate touching the surface of the table. Up to 3 μl of each sample is applied to the center of the membrane bottom of the individual unit of QDB plate in triplicate.
5. **Drying the plate**: The loaded QDB plate is allowed to dry either at room temperate for 1h or at 37℃ for 15 mins in a well-ventilated space.
6. Dried QDB plate is dipped in transfer buffer briefly by gently shaking for 10 sec.
7. QDB plate is rinsed gently with TBST for three times before it is washed for 5min with TBST by constant shaking.
8. **Blocking**: QDB plate is blocked with blocking buffer for 1h with constant shaking.

9. **Primary antibody incubation:** Primary antibody is diluted in blocking buffer at appropriate concentration (from 1: 500 to 1:5000), and aliquoted to individual well of a 96 well plate at 100 μl/well. QDB plate is inserted into the 96 well plate and incubated either for 2 hours or overnight by constant shaking. Alternatively, the incubation step can be done in a box by adding TBST 2 to 3 mm above the membrane portion of the plate if same antibody is used for the whole plate.
10. The plate is rinsed briefly with TBST before it is washed with TBST for three times, each time for 5 mins by constant shaking.
11. **Secondary Antibody incubation**: Secondary antibody is diluted in blocking buffer at appropriate concentration (from 1:1000 to 1:50000), and either aliquoted into a 96 well plate at 100μl/well or in a box, and QDB plate is placed inside either the loaded 96 well plate or the box for 1 hours by constant shaking.
12. QDB plate is rinsed gently three times with TBST, then washed 3 times, 5 mins each with TBST by constant shaking.
13. **Quantification**.
    1. Prepare ECL substrate by following the manufacturer's instructions.
    2. Aliquot ECL substrate into a 96 well plate at 100ul/well, and insert the QDB plate inside 96 well plate for 2 mins.
    3. The plate is removed from 96 well plate, shake briefly to remove the excess liquid, and placed inside a **white** microplate (non-transparent).

4. Turn on the microplate reader, and select “plate with cover” on the user interface before placing the combined plates (QDB plate + white plate adaptor) inside the microplate reader for quantification.
